# Accelerating implant RF safety assessment using a low‐rank inverse update method

**DOI:** 10.1002/mrm.28023

**Published:** 2019-09-30

**Authors:** Peter R. S. Stijnman, Janot P. Tokaya, Jeroen van Gemert, Peter R. Luijten, Josien P. W. Pluim, Wyger M. Brink, Rob F. Remis, Cornelis A. T. van den Berg, Alexander J. E. Raaijmakers

**Affiliations:** ^1^ Computational Imaging Group for MRI diagnostics and therapy Centre for Image Sciences UMC Utrecht Utrecht The Netherlands; ^2^ Department of Biomedical Engineering Eindhoven University of Technology Eindhoven The Netherlands; ^3^ Circuit & Systems Group of the Electrical Engineering Delft University of Technology Delft The Netherlands; ^4^ C. J. Gorter Center for High Field MRI Leiden University Medical Center Leiden The Netherlands; ^5^ Department of Radiology UMC Utrecht Utrecht The Netherlands

**Keywords:** FDTD, implant safety, minimization problems, RF Safety, simulations

## Abstract

**Purpose:**

Patients who have medical metallic implants, e.g. orthopaedic implants and pacemakers, often cannot undergo an MRI exam. One of the largest risks is tissue heating due to the radio frequency (RF) fields. The RF safety assessment of implants is computationally demanding. This is due to the large dimensions of the transmit coil compared to the very detailed geometry of an implant.

**Methods:**

In this work, we explore a faster computational method for the RF safety assessment of implants that exploits the small geometry. The method requires the RF field without an implant as a basis and calculates the perturbation that the implant induces. The inputs for this method are the incident fields and a library matrix that contains the RF field response of every edge an implant can occupy. Through a low‐rank inverse update, using the Sherman–Woodbury–Morrison matrix identity, the EM response of arbitrary implants can be computed within seconds. We compare the solution from full‐wave simulations with the results from the presented method, for two implant geometries.

**Results:**

From the comparison, we found that the resulting electric and magnetic fields are numerically equivalent (maximum error of 1.35%). However, the computation was between 171 to 2478 times faster than the corresponding GPU accelerated full‐wave simulation.

**Conclusions:**

The presented method enables for rapid and efficient evaluation of the RF fields near implants and might enable situation‐specific scanning conditions.

## 1 INTRODUCTION

The group of patients with medical implants that require an MRI scan is constantly growing. However, MRI scanning of a patient with metallic implants bears a potentially severe safety risk. The electromagnetic (EM) fields produced by an MRI scanner can couple to the metallic implant resulting in image degradation and serious health hazards. The largest risk is tissue heating due to the radio frequency (RF) fields. The implant can locally enhance the RF fields causing temperature hotspots^1^, ^2^ with potentially severe consequences.^3^, ^4^ Therefore, people with an implant are either exempted from MRI scanning or scanned with very conservative RF power limitations degrading the achievable image quality severely.

In order to quantify the risks associated with a particular implant EM simulations are often performed. These EM simulations can compute the RF fields for a given transmit coil, patient model, and implant. The electrical properties, conductivity and permittivity, of both the patient^5^ and implant^6^, ^7^ affect the resulting RF fields. The geometry and location with respect to each other of the transmit coil, patient model, and implant are relevant for assessing the RF fields. Finally, the drive settings for the transmit coil are required to correctly quantify the RF fields.^8^, ^9^, ^10^, ^11^, ^12^, ^13^ The simulated RF fields are often used in thermal simulations to quantify tissue heating.

A popular method for EM simulations is the finite‐difference time‐domain (FDTD) method.^14^ With the FDTD method a single configuration of source, patient and implant can be computed at a time. These FDTD simulations are time‐consuming due to the large domain (the whole MRI RF system) that needs to be simulated, even though the implant only affects a small domain within the patient. On top of this, hundredths of thousands of these FDTD simulations, for all the different possible configurations, are required to obtain a conditional label for an implant with the most lenient restrictions on scanning (i.e. the tier 4 approach as specified by ISO/TS 10974:2018^15^). For this reason, investigating RF safety for a particular implant in a patient model is a computationally demanding task. This has been demonstrated by B. Guerin et al^16^ recently for different deep brain stimulation implants. The full‐wave simulation, performed with the finite element method, took up to 6 hours with 13 processors and ∼300 GB of RAM for a single simulation. For FDTD simulations, E. Cabot et al^17^ showed that similar types of simulations can take up to 43 hours for a single simulation, even with GPU acceleration.

To alleviate the computational complexity substitute models are used. One such model used for EM simulations including implants is the Huygens’ box.^18^, ^19^ This method takes a two‐step approach to compute the RF fields. First, a simulation without an implant is done where the RF fields are computed and used to construct a box around the implant. Surface currents running on the Huygens’ box are computed that create the same incident RF field inside this box. After this, the implant is placed inside the Huygens’ box and the surface currents found are applied, on its boundaries, to the simulation which results in the total RF fields. Everything outside of the Huygens’ box is ignored in this second simulation resulting in a smaller computational domain. A challenge with this is making the Huygens’ box large enough such that the scattered RF fields created due to the implant are not reflected back into the box by something that is outside of the box (i.e. there should be no crosstalk between the two domains).

Another substitute model that is applicable to elongated implants, e.g. pacemaker leads, is the electric field transfer function (TF). This transfer function describes the electric field enhancement at the tip of an elongated implant for a given incident tangential electric field exposure,^20^ where the incident tangential electric field is acquired by an FDTD simulation without the implant geometry present. This effectively entails that the scattered RF field due to the implant is superimposed on the incident field, thereby decoupling the concurrent simulations of transmit coil, human model and implant into concurrent simulations with only the transmit coil and human model.

The use of a TF drastically decreases the number of full‐wave simulations that need to be performed. However, as mentioned before, the transfer function is only valid for elongated implants, which is a subset of a large number of different possible implants. Furthermore, the TF can only predict the electric field enhancement at the tip of the implant. The idea of the transfer function was extended to a transfer matrix in the work of J. P. Tokaya et al.^21^ The transfer matrix can predict the electric field enhancement at any location along the elongated implant, rather than only at the tip.

Although the TF enables quick calculation of the RF field enhancement due to an elongated implant, its use comes at the price of a loss of accuracy compared to a full‐wave simulation. This was shown by E. Cabot et al^22^ where it was found that there is a difference (up to 48%) between the concurrent full‐wave simulation of the implant and the patient model compared to computing the response of the implant by the use of the TF.

Due to the aforementioned problems with the current methods, very long simulation times or sub‐optimal accuracy of substitute models, there is a need for a new and more efficient method. Therefore, in this work, we will investigate a fast and accurate generalized methodology to do RF safety assessment for arbitrary implant geometries. This is derived from the work of J. van Gemert et al^23^ that describes a method for efficiently computing the scattered RF fields produced by dielectric pads. Here we use the same methodology for medical implants, thus, instead of computing the EM response of an object near the patient we are interested in the EM response of an object (i.e. an implant) within the patient.

In this method, the RF fields are simulated without the implant present and the scattered RF field produced by the implant is computed and afterward superimposed onto the incident field. The computation to include the EM response of the implant is achieved through a small domain inversion, using the Sherman–Morrison–Woodbury formula.^24^ We assume that the matrix that needs to be inverted is non‐singular, which is normally satisfied.^23^ The inversion is computed on a much smaller domain than the initial simulations. Therefore, the effect of the implant can be computed almost instantly. Furthermore, since the simulation with the source and patient is decoupled from the implant, the electrical properties, geometry, and location of the implant can easily be altered without doing another full‐wave simulation, making this an efficient method for a tier 4^15^ safety assessment.

To compute the scattered RF field due to an implant, a library and the RF fields without the implant are required. The library consists of the EM response for a unitary current density for each location (i.e. voxel edge) that can be occupied by an implant, which can, for example, be simulated using the FDTD method. Computing this library is a one‐time effort and once available it facilitates computing the effect of different materials (i.e. electrical properties) and locations of these implant geometries within the patient can be computed in an extremely fast manner. This decreased computational effort allows for efficient evaluation of the RF safety assessment for implants.

In comparison to existing full FDTD simulations, the presented method achieves unprecedented acceleration factors. This may enable RF safety assessment of implants at much lower costs, may benefit the design of implants, and ultimately may even enable online RF safety assessment of implants.

## 2 THEORY

We follow similar steps as^23^ and start with the Maxwell equations, given by

(1a) $$-\nabla \times H+\sigma E+j\omega \varepsilon E=-J^{\mathrm{ext}},$$

(1b) ∇×E+jωμH=0,

here *H* is the magnetic field, *E* is the electric field, σ is the conductivity, *ɛ* is the permittivity, ω is the angular frequency at the Larmor frequency, μ is the magnetic permeability, and $J^{\mathrm{ext}}$ is the external current density, i.e. the current running through the RF coil. In an MRI environment, all materials have a magnetic permeability of $\mu \;=\;\mu_{0}$. Equations 1a and 1b are defined on a continuous domain. For numerical analysis the domain is typically discretized into a voxelized grid. The discretization of Equations 1a and 1b can be written in matrix vector notation as

(2) $$\left(D+C^{\mathrm{bg}}\right)f^{\mathrm{bg}}=-q,$$

where *D* contains the curl operators and $C^{\mathrm{bg}}$, the electromagnetic properties matrix, contains the electrical properties and is defined as $C^{\mathrm{bg}}\;=\;\text{diag}\left(c^{\mathrm{bg}}\right)$, where the vector $c^{\mathrm{bg}}$ is written as

(3) $$c^{\mathrm{bg}}=\left[\begin{array}{c} \sigma_{1}+j\omega \epsilon_{1}\\ \vdots \\ \sigma_{k}+j\omega \epsilon_{k}\\ j\omega \mu_{1}\\ \vdots \\ j\omega \mu_{l} \end{array}\right],$$

where *bg* is used as shorthand notation for background, indicating that there is no implant present. The subscripts *k* and *l* indicate the number of edges and faces of the discretized domain respectively. The vector $f^{\mathrm{bg}}$ contains the *E* and *H* fields and *q* contains the external current densities. Equation (3) can be written more compact as

(4) $$Af^{\mathrm{bg}}=-q,$$

where $A\;=\;(D\;+\;C^{\mathrm{bg}})$. Solving the field distributions for a given external current density is performed through the inversion of *A*

(5) $$f^{\mathrm{bg}}=-A^{-1}q.$$

It should be noted that *A* encompasses the complete simulation domain, which can be dozens of millions of voxel edges for realistic situations. Therefore, this inversion is not feasible and the fields can only be computed using numerical methods (e.g. FDTD or FEM).

However, we are now interested in a small perturbation in this *A* matrix created by a change in the dielectric properties, for example, due to an implant.

If we were to add this implant to the simulation domain and keep the discretization the same, we would need to solve

(6) $$\begin{array}{cc} (D+C^{\mathrm{bg}}+C^{\mathrm{imp}})f & =-q,\\ (A+C^{\mathrm{imp}})f & =-q, \end{array}$$

where $C^{\mathrm{imp}}$ contains the change in electrical properties between the newly added implant and the background. Similar to Equation (3) we write $C^{\mathrm{imp}}\;=\;\text{diag}\left(c^{\mathrm{imp}}\right)$, where $c^{\mathrm{imp}}$ is defined as

(7) $$c^{\mathrm{imp}}=\left[\begin{array}{c} \sigma_{1}^{\mathrm{imp}}-\sigma_{1}^{\mathrm{bg}}+j\omega \left(\varepsilon_{1}^{\mathrm{imp}}-\varepsilon_{1}^{\mathrm{bg}}\right)\\ \vdots \\ \sigma_{k}^{\mathrm{imp}}-\sigma_{k}^{\mathrm{bg}}+j\omega \left(\varepsilon_{k}^{\mathrm{imp}}-\varepsilon_{k}^{\mathrm{bg}}\right)\\ j\omega (\mu_{1}^{\mathrm{imp}}-\mu_{1}^{\mathrm{bg}})\\ \vdots \\ j\omega (\mu_{l}^{\mathrm{imp}}-\mu_{l}^{\mathrm{bg}}) \end{array}\right],$$

where the superscript *imp* is used as shorthand notation for implant. This operation is equivalent to deleting the electrical properties of the background and adding those of the implant. Since the magnetic permeability of objects in an MRI should be, approximately, equal to $\mu_{0}$ the bottom half of the vector in Equation (7) is equal to zero. Furthermore, at locations where the implant is not present the change in conductivity and permittivity is zero too. Therefore, the change in the medium property matrix is confined to a very small (low‐rank) domain within the matrix *A*. This small domain consists of *M* edges whereas the entire domain on which *A* is defined has *N* edges.

**Figure 1:**
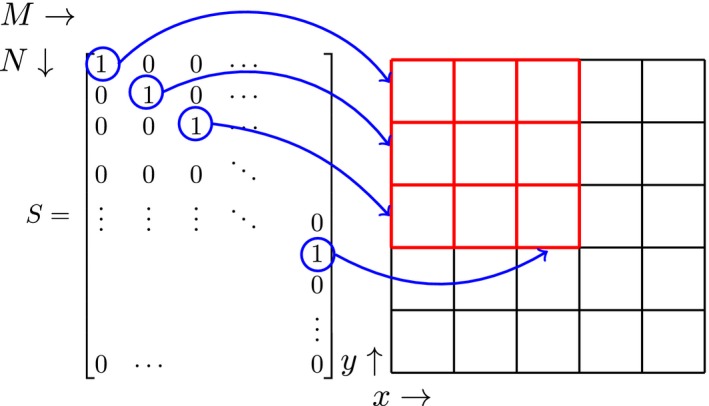
A representation of the *S* matrix for a 2D grid. The left shows the values inside the support matrix for the corresponding edges in the grid on the right. The red edges define the small domain while the red plus the black edges define the large domain. The blue arrows indicate to which edge in the grid each ‘1’ corresponds to

To map quantities from this large domain to the small domain the support matrix *S* is introduced. The *S* matrix has *N* by *M* entries where,

(8) $$S_{\mathrm{ij}}=\left\{\begin{array}{cc} 1, & \text{if}\;{\left(x_{N},\;y_{N},\;z_{N}\right)}_{i}={\left(x_{M},\;y_{M},\;z_{M}\right)}_{j}\\ 0, & \text{otherwise} \end{array}\right.,$$

here *i* and *j* indicate the row and column numbers respectively. Furthermore, ${\left(x_{N},\;y_{N},\;z_{N}\right)}_{i}$ and ${\left(x_{M},\;y_{M},\;z_{M}\right)}_{j}$ describe the coordinates of the $i^{\mathrm{th}}$ edge within the large and $j^{\mathrm{th}}$ edge within the small domain, respectively. Effectively, this entails that there are *M* nonzero entries that indicate when an edge in the large domain coincides with an edge in the small domain as shown in Figure 1. To go from the large domain to the small domain we use

(9) $$S^{T}C^{\mathrm{imp}}S={\tilde{C}}^{\mathrm{imp}},$$

where ${\tilde{C}}^{\mathrm{imp}}$ now describes a diagonal M by M sparse matrix with the values of the electrical properties for each voxel edge occupied by the implant as described by Equation (7). To go from the small domain to the large domain, we use

(10) $$S{\tilde{C}}^{\mathrm{imp}}S^{T}=C^{\mathrm{imp}}.$$

Substituting Equation (10) into Equation (6). and solving for the field distributions results in

(11) $$f=-{\left(A+S{\tilde{C}}^{\mathrm{imp}}S^{T}\right)}^{-1}q.$$

Solving this still requires an inverse operation on the large domain. This is not possible for realistic simulation domains due to the number of edges in the simulation domain. However, there is a matrix identity that allows us to rewrite this equation to our advantage. This is called the Sherman–Morrison–Woodbury matrix identity^24^ and is given for Equation (11) by

(12) $$f=-A^{-1}q+A^{-1}S{\left(I_{M}+{\tilde{C}}^{\mathrm{imp}}S^{T}A^{-1}S\right)}^{-1}{\tilde{C}}^{\mathrm{imp}}S^{T}A^{-1}q,$$

where $I_{M}$ is an *M* by *M* identity matrix. We will now introduce a new matrix called the library matrix *Z*

(13) $$Z=-A^{-1}S.$$

This matrix is an *N* by *M* matrix where every column is the field response for a unitary current density of the corresponding edge in the support matrix *S*. This matrix needs to be simulated before computing the response of any implant. Building the library matrix poses an extensive simulation effort, *M* numerical simulations need to be performed. However, each separate simulation converges quickly because there is only a single edge source present. The library only needs to be computed once for a given dielectric background environment (e.g. for a specific patient model). After this, the response of any arbitrary implant can be calculated almost instantly.

Now substituting Equations (5) and (13) into Equation (12), we obtain

(14) $$f=f^{\mathrm{bg}}+Z{\left(I_{M}-{\tilde{C}}^{\mathrm{imp}}S^{T}Z\right)}^{-1}{\tilde{C}}^{\mathrm{imp}}S^{T}f^{\mathrm{bg}}.$$

Note that the inverse in this equation only needs to be computed on the small domain. This allows the computation of the *E* and *H* to be possible with this methodology. From Equation (14), two key points can be observed. The first is that the total field is the sum of the two different RF fields, the incident fields and a field that is dependent on the scatterer (i.e. the implant). The second is that a generalized form of the transfer matrix^21^ is defined within this equation. This can be seen when realizing that the library matrix, *Z*, has to be multiplied by the scattered current density within the implant. Furthermore, we can observe from Equation 1a that the conductivity and permittivity, the quantities we are changing, are only multiplied with the electric field within $f^{\mathrm{bg}}$. Therefore, the terms in between *Z* and $f^{\mathrm{bg}}$ must be equivalent to the transfer matrix. This point is explained in more detail in Appendix A. The generalized transfer matrix can help provide insight into what implant characteristics significantly influence the scattered RF field. The above‐mentioned key points show another way of looking at how and why this low‐rank inverse computation works and more specifically which electromagnetic quantities affect the total RF field.

Within Equation (14) all the interactions between the electric field components and the resulting current density are defined. Whereas the transfer matrix only uses the $E_{z}^{\mathrm{inc}}$ component of the electric field and results in only the $I_{z}$ component of the current.^21^ This generalized transfer matrix could compute the current running on any arbitrary implant for any incident electric field.

## 3 METHODS

In order to compute the scattered RF field created by an implant using the presented method, a simulation with the transmit coil and patient model is required (i.e. the implant is not present). The RF field computed in this simulation represents the incident/background RF field, $f^{\mathrm{bg}}$. Further, the library matrix, *Z*, needs to be computed. The columns of the library matrix represent the RF fields on the edges in the large domain for a unitary current density, $J\;=\;1A/m^{2}$. All the edges that can be occupied by the implant need to be simulated. Therefore, constructing the library matrix requires a considerably large set of simulations. The described inputs have been computed using an FDTD software package (Sim4Life, ZMT, Zurich, Switzerland).

To validate the method, a separate simulation is performed with the transmit coil, patient model, and implant present. This simulation will produce the total electric and magnetic fields, *f*, which are compared to the total fields produced by the computation performed with the presented method.

**Figure 2:**
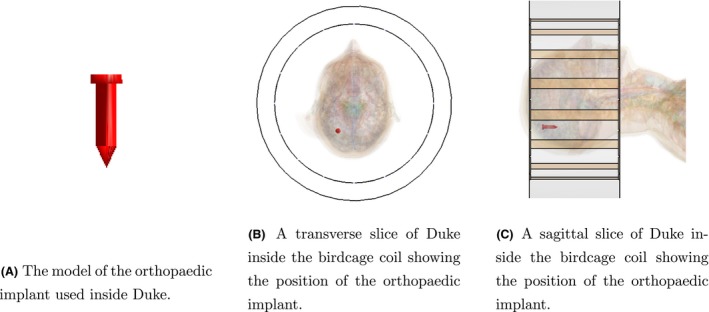
The geometry and location of the passive implant inside Duke

**Figure 3:**
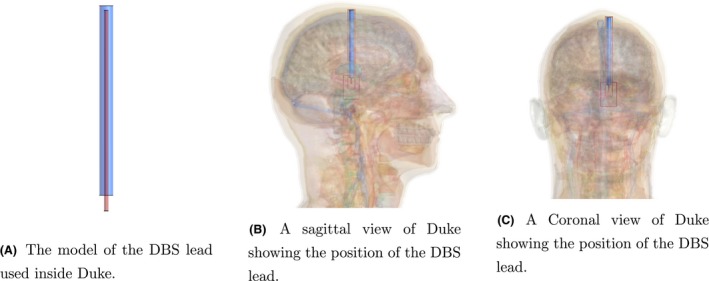
The geometry and location of the DBS lead inside Duke. Duke's position inside the birdcage coil is the same as for the setup with the orthopaedic implant

Three different implant structures are used to test the method. The first represents an orthopaedic surgical implant: a metallic screw. The geometry of the orthopaedic screw is shown in Figure 2A, while the location with respect to Duke and the birdcage coil in the transverse and sagittal plane are shown in Figure 2B,C, respectively. The second implant resembles a deep brain stimulator (DBS) lead structure and the third is a DBS lead structure that is tilted with respect to the FDTD grid axes. Both of these DBS lead structures are shown in Figure 3.

The FDTD simulations for the passive implant are simulated at 128 MHz (3T) and for the DBS implants the simulations were done at 298 MHz (7T). For all implant types, the convergence level of the simulation with and without implant was set at −50 dB, while the library matrix simulation had a convergence level of −30 dB.

The simulations for all three implants were calculated on a 1mm isotropic grid. The orthopaedic screw was simulated with a conductivity of $2.38\cdot 10^{6}\;\text{S/m}$, the conductivity of titanium, and a relative permittivity of 1. The DBS electrode consists of two different materials, a conductive lead and an insulation layer around the lead. For the lead $2.38\cdot 10^{6}\;\text{S/m}$, and $\varepsilon_{r}\;=\;1$ was chosen, while for the insulation material the electrical properties were chosen to be σ = 0 S/m and $\varepsilon_{r}\;=\;4$.

The computations for the FDTD simulations were calculated using a GPU, NVIDIA TITAN X. The update was performed with the Julia programming language^25^ using a CPU, Intel Xeon E3‐1270 v5 (@ 3.60 GHz), and 64GB of RAM available. To solve the inverse in Equation (14), the generalized minimal residual method (GMRES) was used.

As a sanity check that GMRES finds the correct solution, we look at the physical interpretation of the solution of the system *Ax* = *b* in our case. As shown in Appendix A, the solution of our system, *x*, is the scattered current density given by

(15) $$J^{\mathrm{sc}}={\left(I_{M}-{\tilde{C}}^{\mathrm{imp}}S^{T}Z\right)}^{-1}{\tilde{C}}^{\mathrm{imp}}S^{T}E^{\mathrm{inc}}.$$

However, we can also write the scattered current density, per definition, as

(16) $$\begin{array}{cc} J^{\mathrm{sc}} & =\left(\sigma^{\mathrm{imp}}-\sigma^{\mathrm{bg}}+j\omega \varepsilon_{0}\left(\varepsilon_{r}^{\mathrm{imp}}-\varepsilon_{r}^{\mathrm{bg}}\right)\right)E^{\mathrm{tot}},\\ & =C^{\mathrm{imp}}E^{\mathrm{tot}}. \end{array}$$

when written in this form, the scattered current density can be computed using quantities from the FDTD simulations for the incident and total RF fields.

## 4 RESULTS

**Figure 4:**
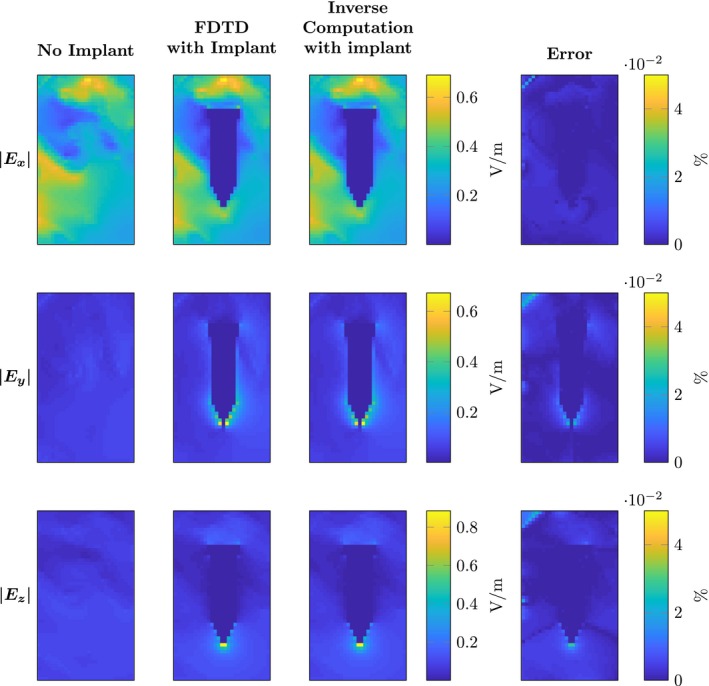
Comparison of the electric field components obtained by FDTD and the proposed inverse computation method from a surgical screw. The three rows show the magnitude of the $E_{x,\;y,\;z}$ components respectively. The first column shows the magnitude of the electric field if there is no implant present. The second column shows the electric fields with the implant present computed by the FDTD method. For the same implant, the third column shows the output of the computations performed with the presented methodology. The last column shows the error percentage as computed by Equation (18)

**Figure 5:**
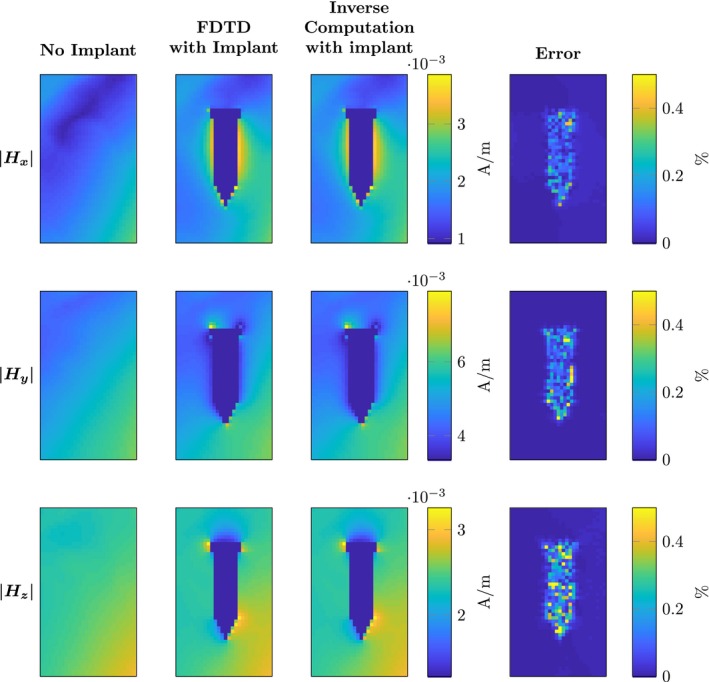
Comparison of the magnetic field components obtained by FDTD and the proposed inverse computation method from a surgical screw. The three rows show the magnitude of the $H_{x,\;y,\;z}$ components respectively. The first column shows the magnitude of the magnetic field if there is no implant present. The second column shows the magnetic fields with the implant present computed by the FDTD method. For the same implant, the third column shows the output of the computations performed with the presented methodology. The last column shows the error percentage as computed by Equation

To validate the presented method, we compare the results from Equation (14) with the simulation from the FDTD solver when the implant is present. For the orthopaedic screw, the results are shown in Figures 4 and 5 for the E and H fields respectively. In both figures, the magnitude of the *x*, *y*, *z* components of the fields is shown. Furthermore, error plots are shown where we defined the error between FDTD fields and the fields as computed by Equation (14) as

(17) $$Err=\frac{\left|f_{\mathrm{FDTD}}-f_{\mathrm{inv}}\right|}{\max(f_{\mathrm{FDTD}})}\cdot 100\%,$$

where $f_{\mathrm{FDTD}}$ and $f_{\mathrm{inv}}$ are substituted for the *x*, *y*, *z* components of the E and H fields, $f_{\mathrm{FDTD}}$ are the fields obtained from the FDTD solver, whereas $f_{\mathrm{inv}}$ denotes the RF fields obtained from the inverse computation. The error is scaled by the maximum value in the field, rather than the local field value. This was performed to suppress errors in regions where the magnitude of the fields are very small (e.g. inside the implant). Otherwise, these small deviations inside the implant would result in large error values although they are of minor concern because the peak values in the electric field contribute significantly more to the heating of the tissue. The ratio between the peak value of the electric field and the electric field inside the implant is a few orders of magnitude. In Table 1, the maximum errors as computed by Equation (17) are shown.

**Table 1:** Maximum error percentage in E and H fields for the passive and DBS electrode

Field component	Orthopaedic screw (%)	Straight DBS electrode (%)	Tilted DBS electrode (%)
$E_{x}$	0.05	1.23	0.57
$E_{y}$	0.04	1.29	0.45
$E_{z}$	0.04	1.25	0.47
$H_{x}$	0.67	1.06	0.26
$H_{y}$	0.62	1.16	0.23
$H_{z}$	1.35	0.37	0.31

**Figure 6:**
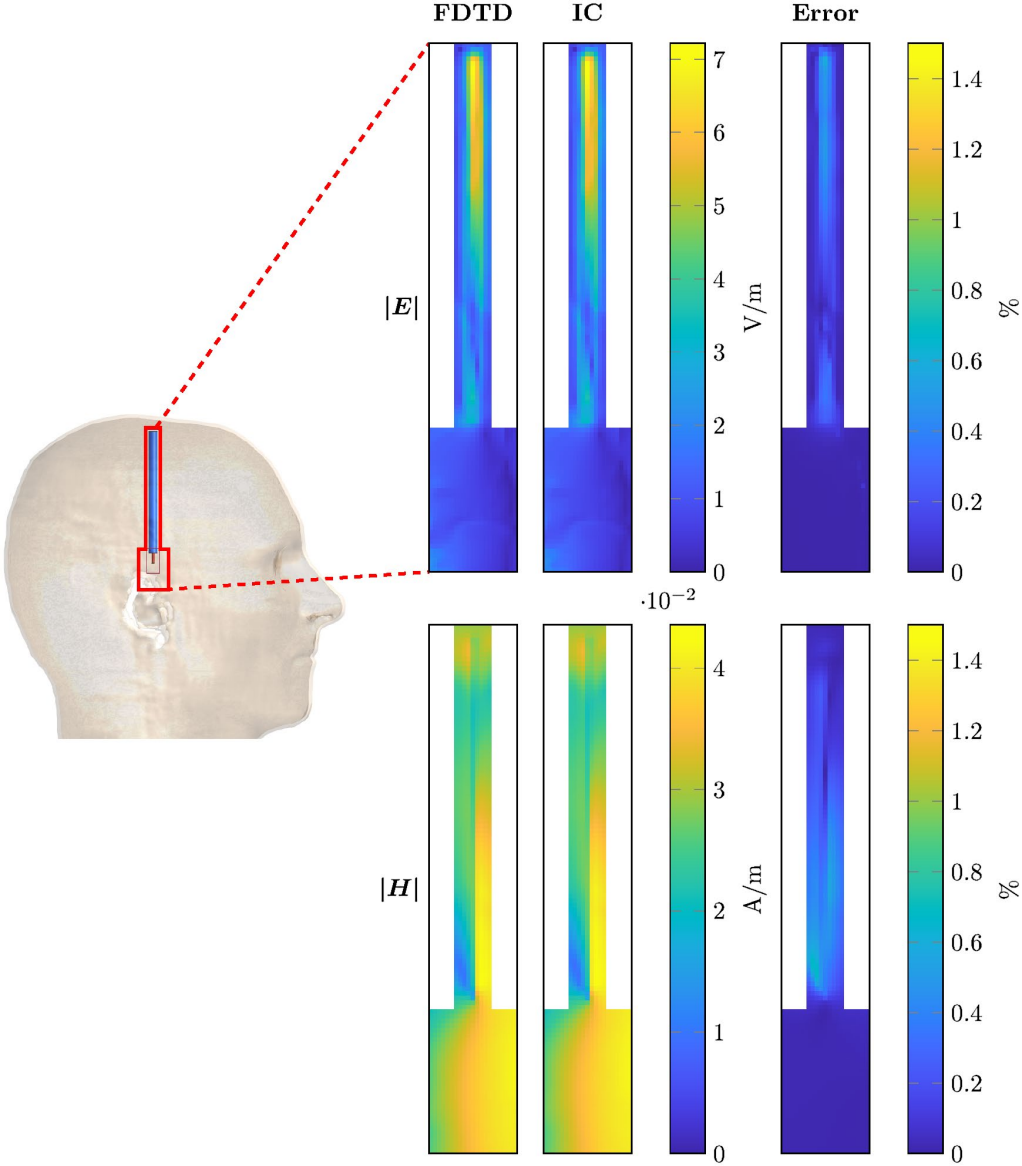
Comparison between the RF fields computed with the FDTD and the presented method for the straight deep brain stimulator lead (aligned with grid axes). On the left, the location of the computed domain within the model is indicated with a red contour. The top row of figures shows the magnitude of the electric field for the FDTD simulation, the inverse computation and the error percentage as computed by Equation (18). Equivalent plots are shown for the magnetic field in the bottom row

**Figure 7:**
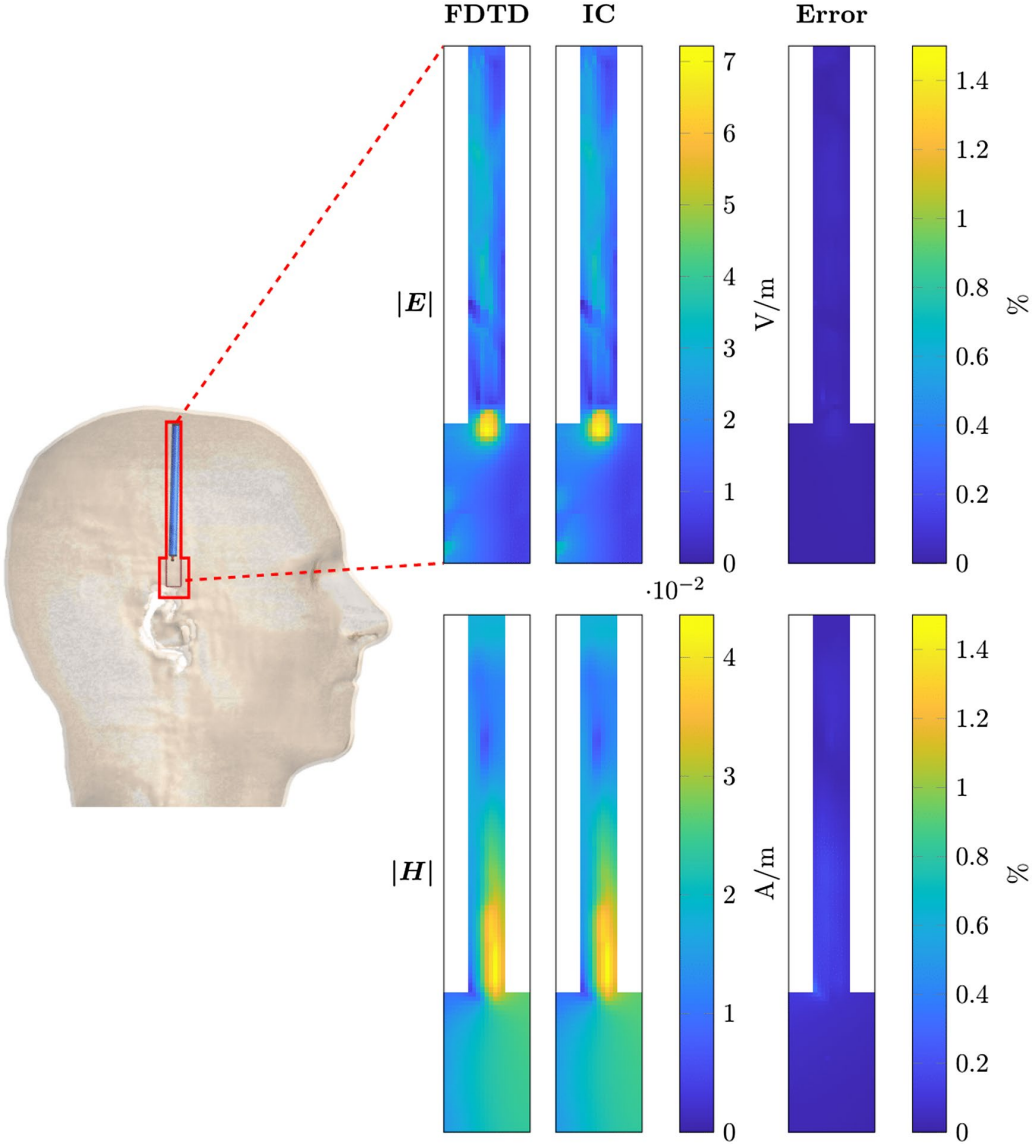
Comparison between the RF fields computed with the FDTD and the presented method for the tilted deep brain stimulator lead (not aligned with grid axes). On the left, the location of the computed domain within the model is indicated with a red contour. The top row of figures shows the magnitude of the electric field for the FDTD simulation, the inverse computation and the error percentage as computed by Equation (18). Equivalent plots are shown for the magnetic field in the bottom row

For the DBS electrodes, the magnitude of the E and H fields are shown in Figures 6 and 7. Again the maximum errors, as defined by Equation (17), are shown in Table 1. Between the three different implants shown, we find that the range of the maximum errors is given by 0.04% to 1.35%.

In Table 2, we compare the dimensions of the problem and the computation time for the FDTD method and the inverse computation. Here the number of edges in the entire domain for the FDTD simulation is given. Furthermore, the number of edges that the implants consist of is shown. This determines both the dimensions of the square matrix that needs to be inverted according to Equation (14) and the number of columns of the library matrix. The computation time, i.e. on the GPU, per column for the library matrix and the total computation time are also given. Finally, the computation times for both methodologies are given together with the acceleration factor. The latter is defined as

(18) $$Acc=\frac{t_{\mathrm{FDTD}}}{t_{\mathrm{inv}}}$$

with *Acc* as the acceleration factor, $t_{\mathrm{FDTD}}$ as the computation time for the FDTD simulation (using either the CPU or GPU) and $t_{\mathrm{inv}}$ for the proposed inverse updating method (CPU based), without the calculation of the library matrix and incident field included. The acceleration factor that is found between the two methods is between 2478 and 171 times faster for the proposed method. This acceleration in simulation time entails that the break even point (BEP) of simulations, meaning that using the proposed method with its corresponding precomputation step is as fast as FDTD, when 22 and 55 simulations are done for the case of the first and second implant, respectively. When more implant geometries/locations with varies incident field exposures are required, which for implant safety assessment standards is certainly the case, the proposed method is faster than FDTD. The BEP is calculated as,

(19) $$BEP=\frac{t_{\mathrm{FDTD}}\;\text{of}\;Z}{t_{\mathrm{FDTD}}\text{(GPU)}-t_{\mathrm{inv}}}$$

**Figure 8:**
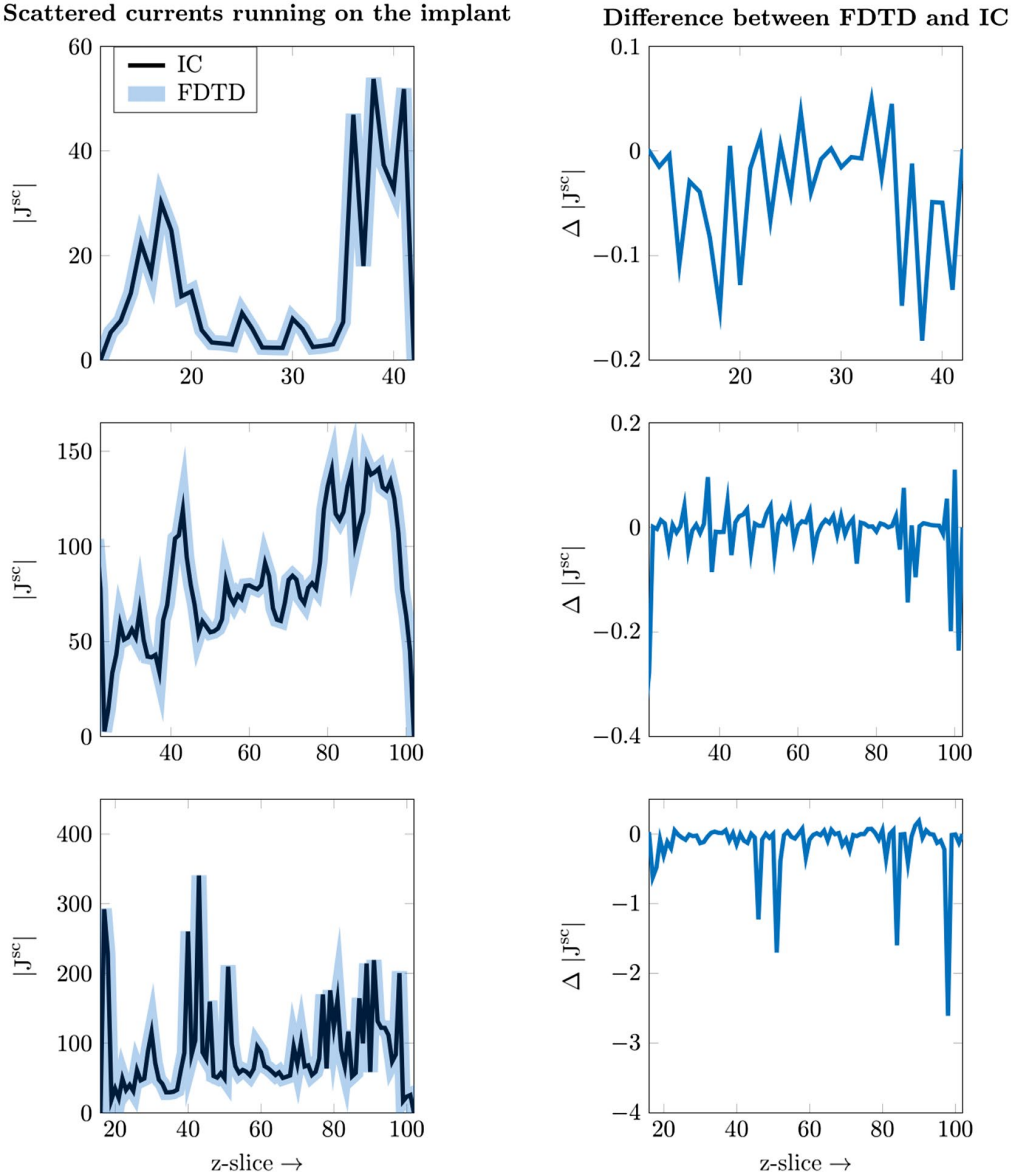
Comparison between the true solution, as computed by Equation (17), and the solution found by the inverse computation, as defined by Equation (16). The current density is summed for the transverse (*xy*‐plane) slices. The top row shows the result for the orthopaedic implant and the second row shows the result for the straight DBS implant and the bottom row shows the result for the tilted DBS lead

Finally, to show that the minimization process finds the correct solution of the system the scattered current density is computed for both implant geometries according to Equations (15) and (16). The current density is summed for all the transverse slices (*xy*‐plane) of the implant to make the plots readable. The result is shown in Figure 8, where it is clearly seen that the minimization process finds the correct solution, i.e. the blue and black line are directly on top of each other and the difference between them is two orders of magnitude smaller than the actual magnitude of the current density.

**Table 2:** Comparison computation time, *t*, between the FDTD method and the inverse computation

	Orthopaedic screw	Straight DBS electrode	Tilted DBS electrode
Total edges FDTD	3.1 $\cdot 10^{6}$	9.9 $\cdot 10^{6}$	9.9 $\cdot 10^{6}$
*N*	1.3$\cdot 10^{5}$	73032	73032
*M*	3804	8794	6583
Length (*z*)	3 cm	9 cm	8 cm
RAM *Z*	8 GB	9 GB	9 GB
$t_{\mathrm{FDTD}}$ for one column of *Z*	20 s	15 s	15 s
$t_{\mathrm{FDTD}}$ of *Z*	21.1 hrs	36.6 hrs	27.4 hrs
$t_{\mathrm{FDTD}}$ (CPU)	14760 s	22790 s	23441 s
$t_{\mathrm{FDTD}}$ (GPU)	3420 s	2414 s	2483 s
$t_{\mathrm{Inv}}$ (CPU)	1.38s	14.1 s	6.8 s
Acceleration (CPU)	10696	1616	3423
Acceleration (GPU)	2478	171	362
Break even point	22.2	54.9	53.2

## 5 DISCUSSION

This work has demonstrated an alternative approach to calculate the RF field response of a medical implant in an MRI. As an input, the method requires the incident RF field distribution (RF field without an implant present) and a library consisting of the unitary current density response of every voxel edge on the discretized implant geometry. To demonstrate the validity of the method, the method is tested for a screw and a deep brain stimulator lead where the input fields are determined by FDTD simulations.

From the maximum errors shown in Table 1, it is clear that this methodology is very accurate. The accuracy is only subject to the numerical precision of the supplied incident and library fields. This is further substantiated by the results shown in Figures 4, 5, 6, 7, 8, where the RF fields and scattered current densities computed by the presented method are shown to be equivalent to those computed by the FDTD method.

One major difference between the presented method and the Huygens’ box is that the reduced domain in the presented method is only as large as the implant itself, whereas with the Huygens’ box the reduced domain should be large enough that there is no crosstalk between the full and reduced domain.

Due to the nature of the inverse computational complexity, $O(M^{3})$, the acceleration factor for this method is dependent on the number of edges that the implant occupies. Therefore, the larger the number of edges the implant occupies the longer the simulation time becomes. This occurs when either the implant size is increased or if the discretization is performed on a finer grid. This is also shown in^23^ and can be observed by Table 2. The computation time of the inverse, however, is independent of the frequency of the RF fields and the voxel size, i.e. geometric resolution, while FDTD simulations are dependent on these properties. This means that very small implants on a very fine grid would require a precomputation step, i.e. computing the library matrix and incident fields, that is slower while computing the EM response of the implant will be equivalently fast for a similar number of edges that need to be updated.

Another, potentially more restricting factor is the memory requirement. The library matrix grows linearly with the number of edges. For the orthopaedic implant given here, the library matrix is already 4GB for the electric fields (and another 4GB for the magnetic fields, however only the electric fields are needed for the computation). On top of this, the memory requirements for the inversion that needs to be computed grows with the square of the number of edges the implant occupies, i.e. 0.1 GB for the orthopaedic implant in this work and 0.6 GB for the DBS electrode. Therefore, while theoretically possible, in the current state of the presented method, it would be difficult to compute the response of highly realistic lead structures. Both due to the large structure of such an implant and the high resolution required to capture all the details, i.e. the helical lead structure. This would increase the simulation time for the incident field and the library matrix. The resulting matrices required for our method would become too large, both *M* and *N* grow cubed with the factor increase in resolution. The memory requirements of the library matrix and the inverse scale with *N* by *M* and *M* by *M*, respectively, i.e. with the $6^{\mathrm{th}}$ power of the factor increase in resolution. We are currently investigating ways to decrease the memory requirements for the presented method. Some of the ideas are discussed below. The current setup and implementation of the presented method would serve well for orthopaedic implants which usually are not tested for RF safety and are either smaller in size or can be modelled on a coarser grid.

To tackle the previously mentioned memory problems, we could approximate the library matrix, *Z*, by exploiting two properties to introduce sparsity into the library matrix. First, the presented method involves the simulation of a full library matrix, while simulations of current density sources that are spatially located near each other have very similar EM responses due to the equivalent dielectric surrounding. Second, the magnitude of the RF fields decays very rapidly for increasing distances away from the source location. This implicates that the value of the current density at any edge of the implant is dominated by the edges that are located close to it. By either interpolating between columns of the library matrix or truncation of the data if the magnitude becomes too small, sparsity can be introduced into the library matrix at the cost of the accuracy of the computation. These and other alterations for improved performance will be investigated in subsequent studies.

Assuming that the limitations described above can be addressed sufficiently, the presented method bears strong potential for applications in RF safety assessment of implants in MRI, since the calculation time of the RF fields is now in the order of seconds. One example is the safety assessment of implants according to the ISO/TS 10974 technical specification. The output of this technical specification is a conditional label for the implant that specifies the maximum $B_{1}^{+,\;rms}$ and/or other RF power‐related settings a patient with the particular implant can safely undergo an MRI examination. For the most rigorous RF safety assessment level (tier 4) of this technical specification concurrent simulations of the implant, patient and transmit coil are required for a wide variety of potential implant locations and trajectories. Although this method will result in the least restrictive scanning constraints, it is often considered too demanding. With the presented method, the field response of every voxel edge in the domain only needs to be calculated once after which the RF field distribution for any potential lead wire trajectory can be computed almost instantly. This may greatly reduce the workload for tier 4 safety evaluations of implants, given that we have access to a library of different RF field exposures and the libraries of different human models.

Another application could be to predict the local RF field enhancement prior to MRI examination of the patient. The implant structure and location could be revealed by the help of previously acquired X‐ray photos of the patient. After the implant is localized a quick RF field calculation could be performed based on pre‐calculated RF field distributions, both for the incident fields and the library matrix, using generic body models. This calculation would result in a situation‐specific power threshold by which the overestimation is reduced to a minimum. This could possibly be achieved for implants without a conditional label, the RF safety assessments could be performed beforehand to verify if a patient with such an implant can safely undergo an MRI exam.

## 6 CONCLUSION

In this work, we have shown a new methodology for RF safety assessment of implants in an MRI setting without assumptions on the implant geometry or composition. With appropriate simulations done beforehand, the presented method can perform the RF safety assessment in a greatly accelerated fashion compared to full‐wave simulations, e.g. FDTD.

The incident fields when no implant is present and a library matrix, containing the EM response of every edge the implant can possibly occupy, need to be precomputed. Afterward, the effect of any arbitrarily shaped and positioned implant, with arbitrary material properties, can be calculated within seconds. The result of the computation is numerically equivalent to the solution of a full‐wave simulation. For the implants shown in this work, the maximum error was 1.35%. However, using this method a significant acceleration is obtained (a factor 171 to 2478 compared to GPU accelerated FDTD simulations). This is excluding the calculation of the library matrix and the incident RF field.

## ACKNOWLEDGEMENT

This work is part of the research programme “Safety threat or measurement device? Using the MRI scanner to assess RF safety of implanted medical devices in MRI.” with project number 15739, which is (partly) financed by the Netherlands Organisation for Scientific Research (NWO).

## APPENDIX A

### A1. Generalized transfer matrix

A generalized form of the transfer matrix as described in^21^ can be extracted from Equation (14). The transfer matrix relates the current in an elongated implant (i.e. a lead wire) with the incident electric field according to

(A1) $$I=ME^{\mathrm{bg}},$$

where *M* is the transfer matrix. The first column of the transfer matrix is the transfer function as defined in.^20^ There are two limitations of these concepts: the first is that TFs are only defined for elongated, linear implants. The second is that they only relate an $E_{z}^{\mathrm{bg}}$ to an $I_{z}$. In an MRI setting, this part of the electric field is the main contributor to $I_{z}$ (which in turn is also the main contributor to the total current *I*). However, the *x*‐ and *y*‐components of the electric field also contribute to the total current that will run on the elongated implant.

The transfer matrix can be obtained from Equation (14) by observing that the library matrix consists of the field responses per unitary current density for each edge of the implant. This implies that to obtain the RF fields produced by the implant a multiplication with the current density at each edge of the implant is needed, which is thus given by

(A2) $${\left(I_{M}-{\tilde{C}}^{\mathrm{imp}}S^{T}Z\right)}^{-1}{\tilde{C}}^{\mathrm{imp}}S^{T}f^{\mathrm{bg}}\equiv J^{\mathrm{sc}}.$$

where $J^{\mathrm{sc}}$ is the current density at each edge of the implant. From Equation 1a, we observe that the dielectric properties in $C^{\mathrm{imp}}$ are only multiplied by the electric field in $f^{\mathrm{bg}}$ and not the magnetic field (i.e. there is no change in $\mu_{r}$). This entails that we can write Equation A2 as

(A3) $$J^{\mathrm{sc}}={\left(I_{M}-{\tilde{C}}^{\mathrm{imp}}S^{T}Z\right)}^{-1}{\tilde{C}}^{\mathrm{imp}}S^{T}E^{\mathrm{bg}},$$

Further we know that the element‐wise multiplication of the area with the current density results in the current as shown by

(A4) I=a∘J,

where *a* is the area of each edge, as defined by the Yee Cell,^14^ of the implant. Using Equation A1 it is now evident that

(A5) $$M=a\;\circ \;\left({\left(I_{M}-{\tilde{C}}^{\mathrm{imp}}S^{T}Z\right)}^{-1}{\tilde{C}}^{\mathrm{imp}}S^{T}\right).$$
